# The Initiation of Swallowing Can Indicate the Prognosis of Disorders of Consciousness: A Self-Controlled Study

**DOI:** 10.3389/fneur.2019.01184

**Published:** 2019-11-14

**Authors:** Jianan Wang, Jing Wang, Xiaohua Hu, Lingqi Xu, Jinna Tian, Jiayin Li, Danruo Fang, Wangshan Huang, Yuxiao Sun, Minhui He, Steven Laureys, Haibo Di

**Affiliations:** ^1^International Unresponsive Wakefulness Syndrome and Consciousness Science Institute, Hangzhou Normal University, Hangzhou, China; ^2^Rehabilitation Center for Brain Damage, Wujing Hospital of Hangzhou City, Hangzhou, China; ^3^GIGA, GIGA-Consciousness, Coma Science Group, Neurology Department, University Hospital of Liege, University of Liège, Liège, Belgium

**Keywords:** disorders of consciousness, swallowing, consciousness, minimally conscious state, prognosis

## Abstract

**Objective:** To detect the initiation of swallowing in patients with disorders of consciousness (DOC) as well as the relationship between the initiation of swallowing and the prognosis of DOC patients.

**Methods:** Nineteen DOC patients were included in this study, and a self-controlled trial compared five different stimuli. The five different stimuli were as follows: (1) one command, as recommended by the Coma Recovery Scale-Revised (CRS-R), which was “open your mouth”; (2) placing a spoon in front of the patient's mouth without a command; (3) placing a spoon filled with water in front of the patient's mouth without a command; (4) one command—“there is a spoon; open your mouth”—with a spoon in front of the patient's mouth; (5) one command, “there is a spoon with water; open your mouth,” with a spoon filled with water in front of the patient's mouth. All 19 patients were given these five stimuli randomly, and any one of the commands was presented four times to a patient, one at a time, at 15-s intervals. The sensitivity and specificity of the initiation of swallowing in detecting conscious awareness were determined.

**Results:** None of the patients responded to the first four stimuli. However, six patients showed initiated swallowing toward the fifth stimulus. Among those six, five patients showed improvement in their consciousness state 6 months later. The sensitivity and specificity of the initiation of swallowing for DOC patients was 83.33% [95% CIs (36%, 100%)] and 92.31% [95% CIs (64%, 100%)], respectively.

**Conclusions:** The initiation of swallowing can be an early indication of conscious behavior and can likely provide evidence of conscious awareness.

**Clinical Trial Registration:** www.ClinicalTrials.gov, identifier: NCT03508336; Date of registration: 2018/4/16.

## Introduction

Disorders of consciousness (DOC) include several states, ranging from coma and unresponsive wakefulness syndrome/vegetative state (UWS/VS) to a minimally conscious state (MCS) (1). Per definition, UWS/VS patients show no sign of consciousness of either themselves or the environment (2). However, MCS patients differ from UWS/VS patients according to the presence of inconsistent but reproducible signs of awareness (3). The clinically heterogeneous MCS patients were subcategorized into two distinct entities: “MCS minus” (MCS−) and “MCS plus” (MCS+) (4, 5). MCS− patients showed low-level purposeful behaviors without command following (e.g., visual pursuit, localization to noxious stimulation, object localization [reaching], automatic motor response, and appropriate smiling or crying related to an external stimuli). MCS plus (MCS+) patients were those who presented higher-level behavioral interactions (e.g., a movement in response to a command, non-functioning communication, and intelligible verbalization). For DOC patients, the differential diagnosis of consciousness state is of great importance, especially for decisions on treatment, care, and end-of-life actions (2, 6).

The assessment of awareness has increasingly been gaining attention and is still an urgent unmet need. At present, the gold standard for diagnosing DOC patients is the standardized behavioral assessment tool (7–9). A misdiagnosis rate of about 40% has been reported by some studies, scilicet some patients with a higher ability were misdiagnosed as being in UWS (10–12). At present, the Coma Recovery Scale-Revised (CRS-R) is strongly recommended and considered for assessing DOC patients (13, 14), with a higher percentage of MCS subjects correctly diagnosed and better overall classification accuracy than the current clinical criteria (12). However, some patient-related factors, such as aphasia, agnosia, cortical deafness, and motor impairment, often lead to a false negative result on a standard CRS-R (7, 15). As has been reported, 19 DOC patients participated in CRS-R and brain–computer interfaces in this study, of which three patients exhibited no responses in the CRS-R assessment but were responsive to auditory startle in the brain–computer interfaces assessment. The results revealed that a proportion of DOC patients who have no behavioral responses in the CRS-R assessment can generate neural responses (16), and a CRS-R total score of 10 or higher yielded a sensitivity of 0.78 for the correct identification of patients in either MCS or EMCS (14). There is an urgent need in behavioral assessment to find effective stimuli to improve diagnostic accuracy; recent studies have shown different stimuli indeed have different effects on the behavioral response of patients (17).

From a recent study, some DOC patients who recovered their swallowing ability at an early stage had a good prognosis (18), and a previous study showed that 64% of DOC patients could recover to unrestricted dieting within 126 days (19). Per another previous report, the initiation of swallowing of the pharyngeal phase is controlled by active cortical control for spontaneous as well as volitional swallowing in awake people (20). The cortex exerts volitional control over the onset and magnitude of neural activity for swallowing. Sensory feedback from the oral cavity, pharynx, and larynx is crucial for initiating the brainstem swallowing response and for modulating cortical activity. Deprivation of sensory input can be detrimental to swallowing safety because it can alter airway protection during swallowing. When peripheral and cortical inputs exceed an activation threshold, the brainstem swallow response is triggered. Additionally, some functional neuroimaging studies have indicated that the left hemisphere has greater activation in certain sensory and motor-related swallowing regions in patients with cerebral vascular accidents (20, 21). Considering that some patients may also have either aphasia or agnosia, some reports have shown that different stimuli have different sensitivity regarding eliciting a behavioral response from DOC patients (22). Hence, the choices of objects and stimuli seem to be important for appropriate clinical behavioral assessment. In this study, we hypothesized that an informative and familiar stimulus might better elicit a response from patients. In addition, we aimed to detect the relationship between the initiation of swallowing and the prognosis of DOC patients using a well-controlled test for the initiation of swallowing for DOC patients.

## Materials and Methods

### Patients

This study protocol was approved by the Ethics Committee of Hangzhou Normal University, which complies with the Code of Ethics of the World Medical Association (Declaration of Helsinki). Written informed consent was obtained from the guardians/next of kin of the patients who participated in the study.

**Figure 1:**
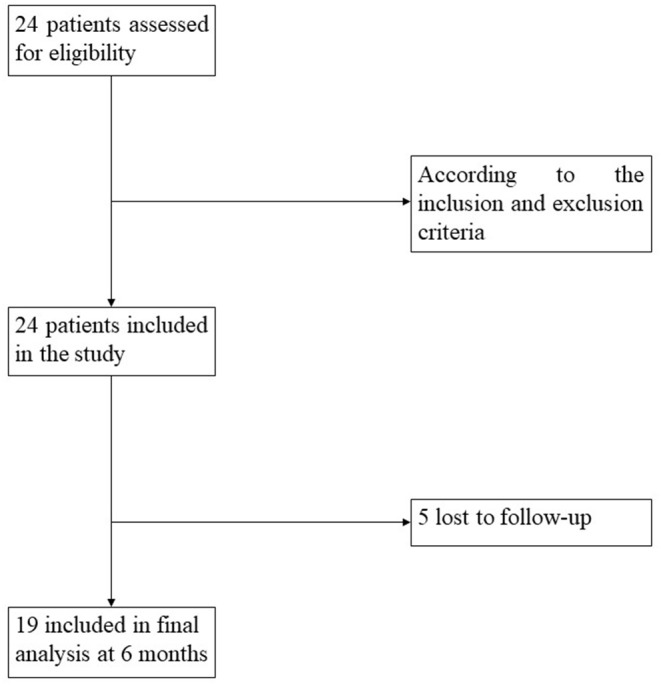
Flow chart of patient selection in the study.

According to the Aspen workgroup criteria for disorders of consciousness (3) and based on the repeated CRS-R assessments, ≥five assessments within 1 week (23) were performed by two trained and experienced neuropsychologists. Meanwhile, each patient's swallowing ability was confirmed by a water drinking test, the protocol of which was as follows: with the patient in a seated position, an injector was used to absorb 30 ml of warm water and fed to the patient. Recorded information was included and recorded the time of drinking water, whether he/she choked or coughed in the process, and whether he/she drank up all the water. We then recruited patients who could drink the water within 5 s in one or two attempts without choking or coughing during the process. In total, 24 patients with the desired swallowing ability who had been diagnosed as either UWS or MCS− were prospectively recruited. Of those 24 patients, 19 (79%) had follow-up information available during the following 6 months (Figure 1). Inclusion criteria included (1) age ≥18 years; (2) no administration of central nervous system stimulants, neuro-muscular blocking agents, or sedatives within the prior 24 h; (3) a diagnosis of UWS or MCS−, based on the behavioral assessment of the standardized CRS-R; (4) periods of eye opening. Exclusion criteria included (1) a documented history of a prior brain injury; (2) a premorbid illness resulting in documented functional disabilities up to the time of injury; (3) acute illness (e.g., pyrexia, pneumonia, or diarrhea); (4) receiving hyperbaric oxygen treatments within 2 h; (5) a fracture of the mandible. The process for recruitment of patients was showed in Figure 1.

### Study Design

Five stimuli were established: (1) one command, as recommended by the Coma Recovery Scale-Revised (CRS-R), which was “open your mouth”; (2) placing a spoon in front of the patient's mouth without a command; (3) placing a spoon filled with water in front of the patient's mouth without a command; (4) one command—“there is a spoon; open your mouth”—with a spoon in front of the patient's mouth; (5) one command, “there is a spoon with water; open your mouth,” with a spoon filled with water in front of the patient's mouth. The patients were placed in a seated position, and we presented the five stimuli in front of each patient's mouth in a random order (i.e., numbers one to five were written (once each) on one of five pieces of paper. We placed the papers into a box, mixed them, drew one, and did not return it to the box, repeating this a total of four times. Therefore, any of the stimuli could be presented each time, one at a time, at 15-s intervals. If the patient opened his/her mouth and attempted to stick out his/her tongue, we considered that the initiation of swallowing had been elicited in that patient.

During this study, the spoon could not touch any part of the patient's body (e.g., mouth, face). Special care was taken not to present stimuli when spontaneous oral movements were occurring. The initiation of swallowing was evaluated through a standardized methodology, as described in the CRS-R (23). Here, we considered that a patient had initiated swallowing if he/she had displayed at least one response to one of the four trials during the presentation of the stimuli. Movements that occurred between stimuli (i.e., after the response interval had elapsed) could not be scored. A complete CRS-R assessment was then performed to diagnose the current state of the patient.

During the assessment, the patients were subject to a standardized arousal facilitation protocol [i.e., we presented deep pressure stimulation unilaterally to the shoulder, arm, and hand until the muscle was firmly grasped at its base between the thumb and forefinger. While squeezing the muscle firmly, it was “rolled” back and forth through the finger tips three to four times (8, 23)].

To obtain a good prognostic value, 6-month follow-up evaluations and further research of the patient's outcomes were conducted via the CRS-R.

### Statistical Analysis

Descriptive data were expressed as median and interquartile ranges (Q) [M (P_25_~P_75_)] for the variables. Differences between the appearances of the initiation of swallowing (positive response), as assessed by five different stimuli, were measured using the Exact Cochran's Q test. The outcome of whether the consciousness state of 19 patients, as assessed by the CRS-R, had improved after 6 months was analyzed by a McNemar's test. We computed the frequency of improvement between the positive and negative reactions during the 6-month follow-up evaluation.

## Results

Demographic and clinical data of the DOC patients who were enrolled in this study are shown in Tables 1, 2. Of the 19 patients (5 females/14 males; age: 57 (49.4~65.6) years; time since injury: 4 (2.36~7.25) months), 11 were diagnosed as MCS−, and 8 were diagnosed as UWS. The etiology was traumatic in 10 patients (e.g., DOC was caused by a car accident, a fall from a high place, etc.) and non-traumatic in 9 patients (e.g., DOC was caused by stroke, anoxia, etc.).

**Table 1:** Demographic characteristics of patients.

**Characteristic**	**Median**	**Range**
Age	57	27–77
Month post-injury	4	1–12
	*n*	
**Gender**		
Males	14	
Females	5	
**Etiology**		
TBI	10	
NTBI	9	

*TBI, traumatic brain injury; NTBI, non-traumatic brain injury*.

**Table 2:** Demographic and clinical data of DOC patients.

**Patient**	**Etiology**	**Time since injury (months)**	**CRS-R (total scores)**	**Sub-scale scores**
MCS-1	TBI	5	10	1-3-2-1-0-3
MCS-2	NTBI	4	10	2-1-3-1-0-3
MCS-3	TBI	1	10	2-3-2-1-0-2
MCS-4	TBI	12	10	1-3-2-2-0-2
MCS-5	TBI	6	10	2-3-2-1-0-2
MCS-6	TBI	8	11	1-3-3-1-0-3
MCS-7	NTBI	4	10	1-3-2-1-0-3
MCS-8	NTBI	3	7	1-2-2-0-0-2
MCS-9	TBI	2	8	1-2-2-1-0-2
MCS-10	NTBI	1	9	1-3-2-0-0-3
MCS-11	NTBI	3	9	0-3-2-1-0-3
UWS1	TBI	6	2	0-0-0-1-0-1
UWS2	NTBI	2	7	2-1-2-0-0-2
UWS3	NTBI	12	7	1-1-2-1-0-2
UWS4	TBI	2	3	0-1-0-0-0-2
UWS5	TBI	10	4	0-0-2-0-0-2
UWS6	NTBI	2	2	0-0-0-0-0-2
UWS7	NTBI	8	4	0-0-2-0-0-2
UWS8	TBI	1	6	1-1-2-0-0-2

*DOC, disorders of consciousness; MCS, minimally conscious state; UWS, unresponsive wakefulness syndrome; CRS-R, Coma Recovery Scale-Revised; TBI, traumatic brain injury; NTBI, non-traumatic brain injury*.

The frequency of the initiation of swallowing that was assessed by different stimuli and the diagnoses at the 6-month follow-up evaluation are listed in Table 3. It shows that none of the MCS− and UWS patients responded to the first four stimuli; however, six DOC patients (five MCS− and one UWS) initiated swallowing toward the fifth stimulus (i.e., a spoon filled with water in front of the patient's mouth and the command “there is a spoon with water; open your mouth”). Two of the five MCS− patients displayed four clearly discernible responses over the four trials, two MCS− patients displayed three clearly discernible responses over the four trials, one MCS− patient displayed two clearly discernible responses over the four trials, and one UWS patient displayed only one clearly discernible response over the four trials. Thirteen patients showed no initiation of swallowing toward any stimuli (Table 3).

**Table 3:** Different responses to different stimuli and the assessment at the 6-month follow-up evaluation.

**Patient**	**Movement to command (positive response frequency/4 times)**					**Diagnosis of CRS-R**	**Diagnosis of CRS-R after 6 months**
	**Only a command**	**Only a spoon**	**A spoon Filled with water**	**A spoon and a command**	**A spoon filled with water and a command**		
MCS-1	0/4	0/4	0/4	0/4	3/4	MCS−	MCS+
MCS-2	0/4	0/4	0/4	0/4	4/4	MCS−	MCS−
MCS-3	0/4	0/4	0/4	0/4	3/4	MCS−	MCS+
MCS-4	0/4	0/4	0/4	0/4	0/4	MCS−	MCS−
MCS-5	0/4	0/4	0/4	0/4	0/4	MCS−	MCS−
MCS-6	0/4	0/4	0/4	0/4	0/4	MCS−	MCS−
MCS-7	0/4	0/4	0/4	0/4	2/4	MCS−	MCS+
MCS-8	0/4	0/4	0/4	0/4	4/4	MCS−	MCS+
MCS-9	0/4	0/4	0/4	0/4	0/4	MCS−	MCS+
MCS-10	0/4	0/4	0/4	0/4	0/4	MCS−	MCS−
MCS-11	0/4	0/4	0/4	0/4	0/4	MCS−	MCS−
UWS1	0/4	0/4	0/4	0/4	0/4	UWS	UWS
UWS2	0/4	0/4	0/4	0/4	1/4	UWS	MCS−
UWS3	0/4	0/4	0/4	0/4	0/4	UWS	UWS
UWS4	0/4	0/4	0/4	0/4	0/4	UWS	UWS
UWS5	0/4	0/4	0/4	0/4	0/4	UWS	UWS
UWS6	0/4	0/4	0/4	0/4	0/4	UWS	UWS
UWS7	0/4	0/4	0/4	0/4	0/4	UWS	UWS
UWS8	0/4	0/4	0/4	0/4	0/4	UWS	UWS

*The first stimulus: only a command (as recommended by the CRS-R); the second stimulus: only a spoon without a command; the third stimulus: a spoon and a command (“There is a spoon; open your mouth”); the fourth stimulus: place a spoon filled with water in front of the patient's mouth without a command; the fifth stimulus: a spoon filled with water in front of the patient's mouth and a command (“There is a spoon with water; open your mouth”). MCS, minimally conscious state; UWS, unresponsive wakefulness syndrome; CRS-R, coma recovery scale-revised*.

**Figure 2:**
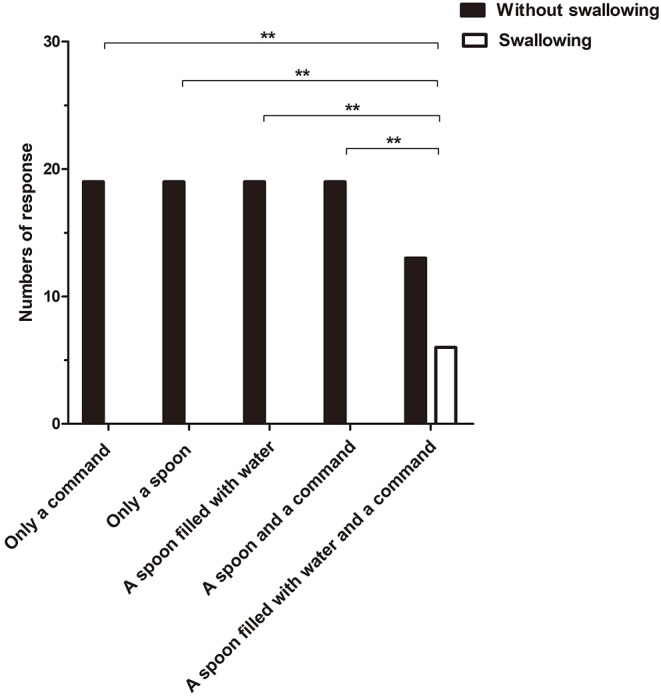
The incidence of the initiation of swallowing to different stimuli differed significantly between the fifth stimulus and the other four stimuli (*Q* = 24, **p < 0.01).

The incidence of the initiation of swallowing differed significantly between the fifth stimulus and the other four stimuli (*Q* = 24, *p* < 0.01) (Figure 2), with response rates of 31.58 and 0%, respectively. In addition, the initiation of swallowing had no significant relationship with either etiology or time since injury (*p* > 0.05).

Six months later, the behavioral follow-up data showed that, of the 19 DOC patients, there had been improvement (i.e., MCS− had turned into MCS+ and UWS had turned into MCS−) in several patients. Among the six patients (five MCS− and one UWS) who had a positive response, five (83.3%) had a good outcome. Of the 13 patients who had no positive response, 12 (92.3%) had a poor outcome. Using the 6-month behavioral follow-up data of the 19 DOC patients for prognostic value statistics, the sensitivity and specificity of the initiation of swallowing for DOC patients was 83.33% [95% CIs (36%, 100%)] and 92.31% [95% CIs (64%, 100%)], respectively (Table 4). The outcome of whether the consciousness state, as assessed by the CRS-R, of the 19 patients had changed (i.e., MCS− had turned into MCS+ and UWS had turned into MCS−) differed, depending on the initiation of swallowing, which was analyzed by a McNemar's test (χ2 = 7.65, *P* = 0.006).

**Table 4:** Prognostic value of initiation of swallowing in DOC patients.

**Initiation of swallowing**	**Initiation of swallowing**	**No initiation of swallowing**	**Total**
Improved at 6 m	5	1	6
No improvement at 6 m	1	12	13
Total	6	13	19

*This study showed the predictive value (sensitivity = 83.33%, specificity = 92.31%) of the initiation of swallowing in DOC patients; five of six (83.3%, [95%CIs (36%, 100%)]) DOC patients who initiated swallowing recovered to either MCS− or MCS+, whereas 12 (92.31%, [95%CIs (36%, 100%)]) of 13 DOC patients with no initiation of swallowing had a poor outcome (remaining in UWS/MCS−). The outcome of whether the consciousness state, as assessed by the CRS-R of 19 patients, had changed (i.e., MCS− turned into MCS+ and UWS turned into MCS−) differed, depending on the initiation of swallowing, which was analyzed by a McNemar's test (χ^2^ = 7.65, P = 0.006, Fisher's exact testing). DOC, disorders of consciousness*.

## Discussion

This study aimed to detect the relationship between the initiation of swallowing and the prognosis of DOC patients. We found that the incidence of patients' movement toward the fifth stimulus (i.e., a spoon filled with water in front of the patient's mouth and the command “there is a spoon with water; open your mouth”) was significantly higher than for the other four stimuli; for high-level behavioral interactions, the initiation of swallowing was more sensitive than the stimuli included in CRS-R. More importantly, the patients who showed a positive response to the fifth stimulus had a higher recovery rate (MCS− to MCS+, UWS to MCS−, as assessed by the standardized CRS-R) (83.3%) at the 6-month follow up. These outcomes support our hypothesis that informative and familiar stimuli may better elicit the response of DOC patients and lead to patients' initiation of swallowing. In addition, the initiation of swallowing can be an early indication of conscious behavior and can probably offer evidence for conscious awareness.

The initiation of swallowing, which can indicate the presence of high-level behavioral interactions in DOC patients, appears earlier than either visual or motor movement, which can indicate the high-level behavioral interactions that are recommended in the CRS-R (e.g., a movement in response to a command, non-functioning communication, or intelligible verbalization). In this experiment, no patients showed either movement to a command, non-functioning communication, or intelligible verbalization, but five MCS− and one UWS initiated swallowing, which indicates a high level of awareness in DOC patients (20). A previous study has shown that the first human reflex is the suckling-swallowing reflex in infants (24). Bremare et al. determined that 7 of 11 (63.6 %) severely brain-damaged patients regained oral feeding abilities after an acquired brain injury (18), and Hansen et al. showed that 64% of DOC patients recovered to unrestricted dieting within 126 days (19). Additionally, some functional neuroimaging studies have indicated that the left hemisphere has greater activation in certain sensory and motor-related swallowing regions in patients with cerebral vascular accidents (20, 21), a study have showed a correlation between the improvement of the swallowing function (i.e., eating solid food safely) and brain neuroplastic changes for the patient with brain injury (25), and some studies have suggested that the management of swallowing disorders, whether they are of either short or long duration, for these patients is important (26–29). Our findings were supported by these studies to some extent, which indicated oral movement may recover more quickly than other functions after brain injury because of neuroplasticity and other reasons and emphasized the importance of oral movement in the process of behavioral assessment and the relationship between the initiation of swallowing and the prognosis of DOC patients. In this way, the initiation of swallowing maybe more appropriate than the stimuli included in CRS-R to trigger high-level behavioral interactions in DOC patients in early stages after injury.

A literature review revealed that our findings seemed to be supported by several studies, which suggested that familiar stimuli have been frequently used to capture a patient's attention. Sharon et al.'s study proved that familiar faces succeed in eliciting activations in brain areas, with further limbic and cortical activations in VS patients (30). Di et al.'s study showed that having family members use a patient's name elicits more responses than a neutral voice does (22, 31, 32). Notably, previous studies have suggested that brain lesions may even lead to receptive aphasia (33), the incidence of which has ranged from 15 to 30% (34, 35). In other words, there are probably some aphasic patients in the present study. However, high frequency and the use of familiar words are easier for these aphasic patients to understand (e.g., “Close your eyes”; “Open your mouth”) (33). Therefore, a gestural or graphical presentation was suggested after a failed verbal item during the assessment process (36). That is, life-familiar stimuli (i.e., feeding water to patients like a newborn baby) might improve the incidence of the initiation of swallowing. In this study, we chose an object from everyday life (i.e., spoon) and gave the specific characteristics of this object (water in the spoon). The fifth stimulus consisted of a verbal request and a gestural presentation; therefore, this stimulus may be better for patients who have a co-occurring language disorder in consciousness and is more suitable for detecting those underlying aphasic patients. From the reaction results, this stimulus improved the incidence of the initiation of swallowing.

In our results, those six DOC patients who initiated swallowing toward the fifth stimulus were five MCS− and one UWS; in other words, the initiation of swallowing can be more easily elicited in MCS− than that in UWS. This result is in line with some literature showing a low level of arousal to be a negative predictor of oral refeeding and the recovery of swallowing function related to the severity of the brain injury. Hansen et al.'s study showed that 64% of DOC patients recovered to unrestricted dieting within 126 days, and the chance of returning to a total oral diet depended on the severity of the brain injury (19). Terre et al.'s study reported the greater the severity of the TBI according to the outcome scales was, the worse the recovery of swallowing function will be. It also noted that improved deglutition function paralleled improved neurological function and, therefore, dysphagia appears to be a manifestation of greater neurological and functional deficits (37). Moreover, Calabrò et al.'s study proved that dysphagia rehabilitation improved cognitive levels in patients with major neurocognitive sequelae following severe brain injury (25). Linda et al.'s study proved that, as certain cognitive levels improved, patients with severe brain injury were able to achieve greater oral intake (38). Therefore, we have reason to doubt that the patients who respond to the fifth stimulus may be misdiagnosed as MCS−, and their correct diagnosis may be MCS+. The cause of this phenomenon might result from the fact that the stimuli recommended by the CRS-R are not so sensitive that patients cannot make a response. Based on these reasons and with improved neurological functions, DOC patients with a positive response to our experiment had a good prognosis after 6 months.

Previous studies have revealed that patients whose etiology was traumatic showed a significantly higher recovery rate than patients whose etiology was non-traumatic (39). In our study, of five patients who had a good outcome, two were traumatic and three were non-traumatic, and the occurrence of the initiation of swallowing had no significant relationship with etiology. On this point, our conclusion seems inconsistent with the previous literature. However, functional magnetic resonance imaging (fMRI) studies have identified anatomic regions that are active during swallowing, including the primary sensory and motor cortex, supplementary motor area, cingulate cortex, insula, operculum, prefrontal and inferior frontal cortex, basal ganglia, thalamus, and cerebellum (21). The use of fMRI has confirmed that neuroplasticity is the mechanism by which the damaged brain relearns “lost behavior” in response to rehabilitation (40). The reason for this phenomenon may be that swallowing is related to many regions of the brain; if some parts are damaged, other parts could compensate to some extent. Therefore, the occurrence of the initiation of swallowing had no significant relationship with etiology, and, perhaps because of this reason, the recovery of swallowing occurs sooner than other physical functions do.

## Study Limitations

For the two MCS− patients, the diagnosis was maintained as MCS−, which may be related to the fluctuations of consciousness level that have been mentioned in the literature (7). Although the findings are intriguing, there are several limitations in this study. The sample included only 19 patients, and the follow-up duration was only 6 months. Further investigation with a larger sample needs to be done to validate our findings.

## Conclusions

In conclusion, this study emphasizes that the initiation of swallowing can be an early indication of conscious behavior and can probably provide evidence of conscious awareness in DOC patients. Meanwhile, this study showed that using familiar things is more effective than general stimuli in capturing DOC patients' attention (30, 41).

## Data Availability Statement

All datasets generated for this study are included in the manuscript.

## Ethics Statement

The studies involving human participants were reviewed and approved by the Ethics Committee of Hangzhou Normal University. The patients/participants provided their written informed consent to participate in this study.

## Author Contributions

JNW, LX, JT, JL, DF, WH, XH, and YS substantially contributed to acquisition of data. JNW, JW, and WH substantially contributed to analysis of data. JNW, HD, SL, and JW substantially contributed to interpretation of data. MH, SL, and HD substantially contributed to study supervision.

### Conflict of Interest

The authors declare that the research was conducted in the absence of any commercial or financial relationships that could be construed as a potential conflict of interest.
